# Understanding Care‐Seeking Behavior for Reproductive Tract Infections Among Afghan Women: A Cross‐Sectional Study

**DOI:** 10.1002/puh2.70072

**Published:** 2025-06-18

**Authors:** Cecilia Acuti Martellucci, Nooria Mohammady, Fawzia Negin, Sayed Hamid Mousavi, Adriana Viola Miranda, Husna Safa, Bibi Qudsia Qasimi, Khaterah Mosavi, Basira Bek, Alisina Azimi, Zahra Annabi, Saddiqa Noorzai, Aabidullah Rahimee, Taha Balaghat, Fatema Rezaie, Fardına Temory, Mirwais Ramozi, Mosè Martellucci, Rahila Bek, Shabanah Noorzai, Madina Niro, Husna Sultani, Palwasha Farooqi, Ehsan Shayan, Mohammadgul Zhwand, Qasem Rezaee, Farzana Torgani, Bibi Shakiba Hussaini, Shaqaiq Akhtiyari, Farah Qaderi, Shohra Qaderi

**Affiliations:** ^1^ Department of Environmental and Prevention Sciences University of Ferrara Ferrara Italy; ^2^ Kabul University of Medical Sciences Kabul Afghanistan; ^3^ Faculty of Medicine Balkh University Balkh Afghanistan; ^4^ Afghanistan National Charity Organization for Special Diseases (ANCOSD) Kabul Afghanistan; ^5^ Materno‐Fetal and Obstetrics Research Unit Department Woman‐Mother‐Child Lausanne University Hospital Lausanne Switzerland; ^6^ Medical Research Center Kateb University Kabul Afghanistan; ^7^ Global Health Focus Asia Bandung Indonesia; ^8^ Jami University Herat Afghanistan; ^9^ Rabia Balkhi Medical Complex Hospital Ministry of Public Health Kabul Afghanistan; ^10^ Massachusetts General Hospital, Harvard Medical School Boston Massachusetts USA

**Keywords:** Afghanistan, reproductive tract infections, women

## Abstract

**Background:**

In Afghanistan, providing care for reproductive tract infections (RTIs) is challenging, among other factors, due to the extreme scarcity of reliable data. To address this gap, the present study investigates symptoms, potential risk factors, knowledge, and care‐seeking behavior in the largest sample of women to date.

**Methods:**

From September 16, 2022 to November 26, 2022, a structured questionnaire was administered to women presenting at multi‐specialist clinics in the major cities of Afghanistan. Signs and symptoms of RTIs were investigated, together with reproductive history, hygiene practices, and sociodemographic characteristics. Logistic regression, adjusted for selected covariates, was used to assess predictors of delays (over 1 month) from symptoms onset to care‐seeking, and of a history of RTI.

**Results:**

A total of 601 responses were analyzed (80.2%). Mean age was 31.3 years (standard deviation [SD] 11.5). Signs symptoms related to RTIs were reported by 79.2%, knowledge of RTIs by only 23.0%, and care‐seeking delays by up to 39.5%. Care‐seeking delays were positively associated with abnormal vaginal discharge (odds ratios [OR] 4.12; 95% confidence intervals (CI) 2.01–8.45), lower abdominal pain (2.62; 1.44–4.77), and fever (1.93; 1.25–2.98) and negatively associated with being sedentary (0.38; 0.22–0.64), hand washing (0.61; 0.40–0.95), and knowledge about RTI, although borderline significant. A history of RTI (reported by 44.1%) was predicted by abnormal vaginal discharge, fever, irregular menstruations, and use of sanitary pads but not by the husbands’ history of RTI.

**Conclusions:**

The majority of women presenting at clinics in Afghanistan reported symptoms related to RTIs, delayed care‐seeking, and lack of knowledge about RTI. Healthcare providers should inform the population about RTIs and their standard care pathway, while adopting a multi‐dimensional approach accounting for the cultural background of the women.

## Graphical abstract

Afghan women face significant challenges to meet their reproductive health needs. To address the gap, this is the first study with a large sample of women from multiple cities in Afghanistan. The majority of our study participants showed symptoms of RTIs, delayed care‐seeking, and lack of knowledge about RTI.

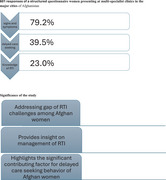

## 1 Introduction

Reproductive tract infections (RTIs) are both preventable and potentially treatable [1], however, they are often asymptomatic or exhibit indistinguishable symptoms, leading to delayed diagnosis and treatment and possibly severe or lethal conditions [2, 3]. RTIs, although rarely comprehensively assessed, tend to be particularly prevalent in low‐ and middle‐income countries: limited education can lead to behaviors that increase the risk of infection, cultural stigma often deters women from seeking medical care, and finally access to healthcare can be severely restricted [4].

In Afghanistan, access to medical care is especially limited for women and children, also due to high transportation and medication costs [5, 6]. Reports indicate that Afghan women receive insufficient healthcare services, are at high mortality risk during pregnancy and childbirth, have an estimated maternal mortality rate of 638 per 100,000 live births [7, 8]. Education and awareness on reproductive health are severely lacking, with prevalent adolescent pregnancies, women bearing an average of seven children, and abortion being authorized only when the mother's life is at risk [9, 10]. Moreover, societal norms dictate that Afghan women have limited decision‐making power, even concerning their own health, which further hampers their chances to receive care [9, 11].

Reliable data on the prevalence of RTIs is severely lacking in Afghanistan, where syphilis was extremely widespread in the 1950's, with an estimated prevalence of about 25% [12]. Only one evaluation by the global burden of disease study provides estimates of the impact of RTIs in 1990 and 2019, and it suggests that although gonococcal and genital herpes infections are decreasing, trichomoniasis and chlamydial infections are slightly increasing, whereas syphilis is swiftly becoming more widespread once again [13]. Given the need of updated data on Afghan women, this work aims to assess symptoms, potential risk factors, knowledge, and care‐seeking behavior among women interviewed at healthcare clinics.

## 2 Methods

### 2.1 Study Aim, Design, and Setting

This was a multicenter cross‐sectional questionnaire‐based study, and it aimed to calculate the prevalence of RTI signs and symptoms among women visiting clinics across Afghanistan, as well as to investigate, among the same women, potential predictors of delayed care‐seeking and of having had RTIs in the past. The study was conducted in 16 clinics (multi‐specialist, both medical and surgical) located in major cities of Afghanistan, including Kabul, Herat, Farah, Badghis, Ghoor, Nimruz, and Parwan. The STROBE checklist was filled [14].

### 2.2 Participants

Eligible women were:

1. aged 16 to 65 years old;
2. able to answer the questions;
3. had provided verbal informed consent;
4. presented spontaneously to the clinic with any type of signs, symptoms, or requests.

The data collection was carried out from September 16, 2022 to November 26, 2022. Of note, the study faced an unexpected delay compared to the initially defined protocol. This delay was a result of a devastating attack in Kabul on September 30, 2022. Many members of our data collection team experienced the loss of their family members during this incident; thus, we had to interrupt the study from September 30, 2022 to October 30, 2022 to provide support and allow for grieving.

### 2.3 Data Collection and Data Entry

The interviews were conducted face‐to‐face by female medical students in the native language of respondents (Pashto or Dari). Kabul female medical students underwent online training (four 2‐h sessions) conducted by two registered psychologists, which was aimed to equip them with the necessary skills and knowledge to conduct the study in a culturally appropriate manner. Upon completion of the training, the students were authorized to begin data collection. Their progress was monitored and assessed on a weekly basis to ensure adherence to the study protocol and to provide support and guidance as needed.

The questionnaire was anonymous, and the pseudonymization of individual records was ensured by associating a numerical ID to all women. The records were generated during the interviews as the medical students input the replies into an online forms, and they were then stored separately from the women's official medical records at all times, with no possibility of associating women to their study data. All data was pooled, through an automatic encrypted online transfer, into an Excel file (Excel 2018, Microsoft Corporation, Washington, USA), which was only accessible to authorized personnel.

### 2.4 Survey Tools

A structured questionnaire was developed in English, drawing from the standard anamnestic questionnaires conventionally used in the clinics where the study was conducted and also from peer‐reviewed studies, in order to maximize comprehensiveness and comparability [2, 9, 10, 15, 16, 17, 18]. Subsequently, a focus group of four gynecology and obstetrics specialists, two generalist medical doctors, and two infectious disease experts reviewed the questionnaire, which was then translated into Pashto and Dari in order to administer it in the native language of participants [19]. Finally, the questionnaire was preliminarily tested on 10 women who met the inclusion criteria, and possible critical points were corrected. These first 10 responses were excluded from the analyses as the questionnaire was subsequently revised, although slightly.

The questionnaire included the following items, pertaining to different main topics:

1. sociodemographic and economic: age, marital status, education, husband's age, occupation, ethnicity, address, women's occupation, and income;
2. anamnesis: past medical history, immunosuppression, and physical activity (categorized as sedentary, moderate, or vigorous according to the World Health Organization (WHO) guidelines);
3. gynecology and obstetrics history: hormone replacement therapy, cervical screening, and signs and symptoms related to polycystic ovary syndrome (including hyperandrogenism, oligomenorrhea, and polycystic ovaries, according to WHO diagnostic criteria);
4. clinical presentation of potential RTIs: signs and symptoms of lower genital tract infections, including vaginal discharge, itching, fever, and lower abdominal pain, occurred in the last month before the interview [2];
5. hygiene practices and attitudes, knowledge and awareness regarding RTIs: hygiene practices during menstruation, overall personal hygiene [10], perception of RTIs, healthcare seeking, and level of information about RTIs and their transmission [9, 20].

### 2.5 Data Analysis

Descriptive statistics were compiled for all characteristics investigated by the questionnaire, showing percentages for categorical variables and means with standard deviation (SD) for continuous ones. Concerning signs and symptoms of RTIs, the prevalence was calculated of women complaining from the following: abnormal vaginal discharge, vaginal itching, smell from genitals, lower abdominal pain, and fever.

Concerning multivariate analyses, the following two outcomes were investigated:

1. delayed care‐seeking, defined as a wait of at least 30 days between signs and symptoms onset and seeking care at an official healthcare provider, for reasons other than mismanagement at a previous clinic;
2. positive history for RTI, defined as at least one self‐reported diagnosis of RTI at any point in the woman's life.

The potential predictors of the two outcomes were investigated using logistic regression, adjusted a priori for the following covariates (all self‐reported): signs and symptoms of RTI, age, marital status, schooling level, knowledge on RTIs, income, physical activity, husband's history of RTI, menstruation irregularities, hygiene practices, use of antibiotics, and condom use. Odds ratios (OR) with their 95% confidence intervals (CI) were calculated, and a rate of 1 covariate to 10 failures was respected to avoid over‐fitting. All tests were two‐tailed, and a *p* value < 0.05 was considered significant. All analyses were conducted using STATA 15.1 (StataCorp. 2017, College Station, TX).

## 3 Results

**FIGURE 1:**
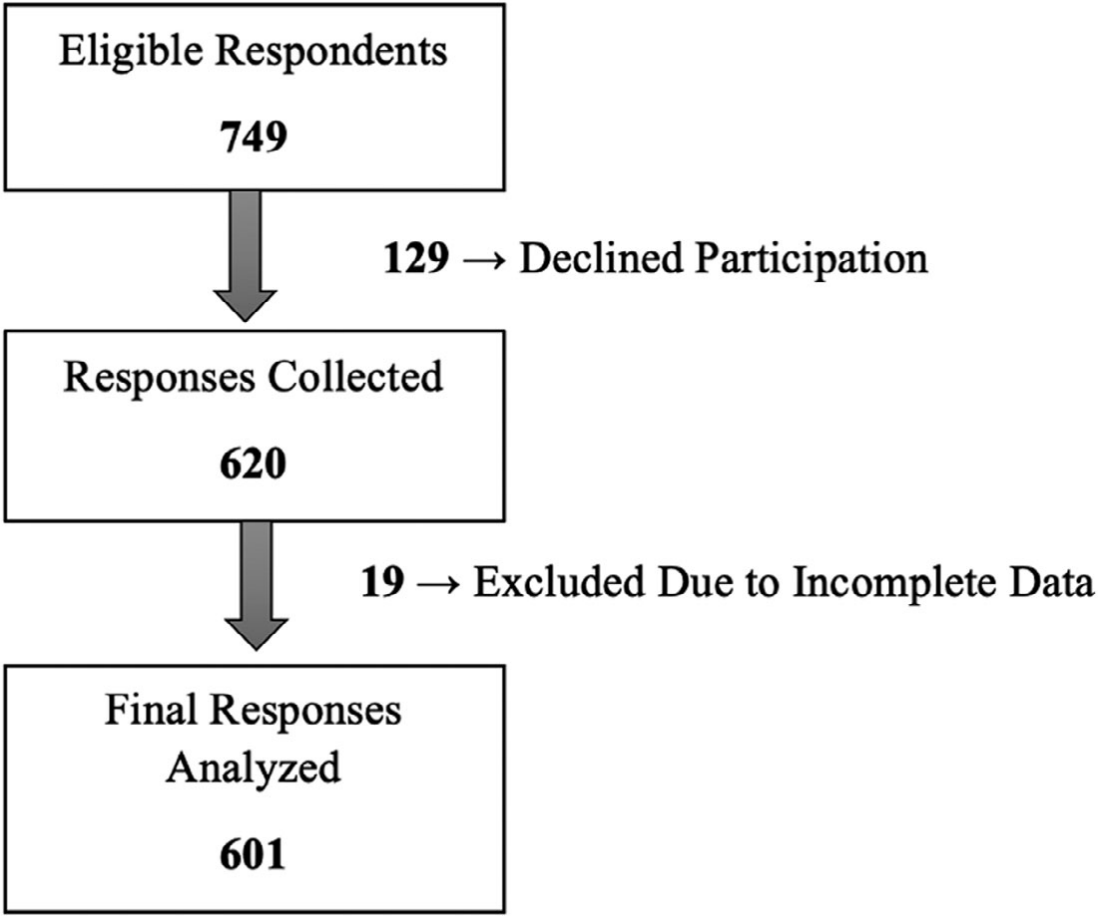
Study participation flowchart: Response rate and data inclusion process.

The study involved the collection of 620 records from respondents, out of 749 who were eligible and were asked to provide their replies (for an 82.8% participation rate). After excluding 19 records due to incomplete data, a total of 601 responses were included in the analysis (final participation rate: 80.2%) (Figure 1).

### 3.1 Sociodemographic Characteristics

The mean age of the participants was 31.3 years (SD—11.5 years), and the majority of study participants (69.1%) were younger than 35 years (Table 1). Among all the participants, with 63.9% were the sole wife of their husbands, whereas 6% were in a polygamous marriage. Regarding educational attainment, a significant proportion of participants were found to be illiterate (32.5%), although 28.6% were university graduates. The occupation of the majority of the women (58.2%) was housekeeping. The majority of the participants reported an average income (62.1%) and resided in urban areas (72.7%). The mean age of the women's husbands was 40 years old (SD 12.3 years), and dysuria and RTI history were reported for, respectively, 12.3% and 8.9% of them (Table S1).

**TABLE 1:** General characteristics of the women (*N* = 601).

Characteristics	Proportions (%)^a^
Age in years, mean (SD)	31.3 (11.5)
Age in years	
<20	9.5
20–25	30.8
26–35	28.8
>35	29.4
NR	1.5
Marital status	
1st wife	3.3
2nd wife	2.8
Only wife	63.9
Single	25.5
Widow	3.2
Other	1.3
Schooling	
Illiterate	32.5
Primary school	18.5
High school	20.1
University	28.6
NR	0.3
Occupation	
Housewife	58.2
Unemployed	25.4
Official employment	8.0
Informal employment	5.2
NR	3.2
Self‐reported income	
Above average	6.0
Average	62.1
Below average	30.4
NR	1.5
Residence	
Main city of the province	72.7
Villages	26.8
NR	0.5
Ethnicity	
Hazara	49.4
Tajik	26.0
Pashtun	19.1
Turkmen	1.5
Uzbek	1.0
Other	3.0
Physical activity	
No	27.3
Yes, less than 2 h per week	35.8
Yes, more than 2 h per week	36.4
NR	0.5
Hormone replacement therapy	12.0
NR	17.0
History of polycystic ovary	20.1
NR	5.2
History of positive HPV test or Pap smear	4.0
NR	6.0
History of allergy	25.1
NR	0.2
Immunosuppression	17.5
NR	0.2
Contraceptive use	
Condom	29.0
Oral hormonal therapy	22.3
Both condom and oral hormonal therapy	10.6
NR	8.5

*Note:* NR, not reported (equals 0.0% when absent).

Abbreviation: SD, standard deviation.

^a^ Percentages unless otherwise specified.

### 3.2 Gynecological Characteristics

The mean age at menarche was 13 years old (SD 1.9 years), with only 10.7% of the sample that reported menopause (Table S2). Among the participants, 65% reported using sanitary pads, whereas 32.2% used cloth materials during menstruation. Irregular menstruation was observed in 32.0% of women, whereas 77.0% experienced painful menstruation.

### 3.3 Reproductive History

The mean age at marriage was 19.5 years (SD 4.2 years), whereas the mean age at first delivery was 21.1 years (SD 3.7 years—Table S3). Over a third of the participants (40.8%) reported having their first delivery between the ages of 17–20 years. Over all 22.0% of the sample reported having more than four children alive, and indeed 25.0% of the women underwent more than four vaginal deliveries, whereas 64.3% did not undergo any C‐sections. The mean time interval between the last two childbirths was 34 months, and 14.8% of respondents were pregnant at the time of the survey. In terms of the place of delivery, 48.9% of the women reported always going to the hospital, 20.0% used both the hospital and the home, and 15.2% only gave birth at home. A total of 18.1% reported being unable to conceive even after an average of 34 months of carefully timed, unprotected sex; however, only 3.7% of respondents reported undergoing in vitro fertilization (IVF—Table S4).

### 3.4 Contraceptive Use

The most frequently reported contraceptive was condom (29.0%), followed by oral contraceptive (22.3%) and their combined use (10.6%—Table 1).

### 3.5 Characteristics Related to Suspected RTI at Last Check‐Up

An abnormal vaginal discharge was reported by a sizeable part of the women (76.5%—Table 2). Of these, the discharge was described as white by 48.3% and as yellow or green by 39.1%. Severe vaginal itching was reported by 29.1% of participants and mild itching by 26.0%. Abdominal pain was reported as constant and spontaneous by 25.9% of the women, as concomitant with menstruations by 49.7%, and as linked to sexual intercourse by 18.0%. A history of RTI was reported by 44.1% of the women, and antibiotic use (including both prescription and over‐the‐counter) was found in 31.1% of participants. Only 23.0% of women had knowledge and awareness regarding RTIs and their transmission.

**TABLE 2:** Characteristics related to suspected reproductive tract infection (RTI) at last check‐up (*N* = 601).

Characteristics	Proportions (%)
Number of signs and symptoms reported^a^	
None (including not reported)	20.8
One	9.2
Two	13.6
Three	17.3
Four	15.8
Five	23.3
Perceived abnormal vaginal discharge	76.5
NR	2.0
Vaginal discharge quantity (*N* = 460)	
Abundant	36.5
Scanty	53.5
Irregular	6.5
NR	3.5
Discharge color (*N* = 460)	
White	48.3
Yellow or green	39.1
Other or no specific color	10.9
NR	1.7
Discharge consistency (*N* = 460)	
Thin	45.4
Thick	42.6
Frothy	7.0
Other	3.3
NR	1.7
Discharge duration (*N* = 460)	
≤6 weeks	38.3
6 weeks–6 months	22.6
7–12 months	35.6
NR	3.5
Vaginal itching	
No	33.1
Severe	29.1
Mild	26.0
NR	11.8
Smell from genitals	
No	40.3
Fishy smell	24.9
Foul smell	20.8
NR	14.0
Lower abdominal pain	75.0
NR	1.3
Timing of the pain (*N* = 451)	
Constant spontaneous pain	25.9
Menstruation	49.7
Sexual intercourse	18.0
Other	2.2
NR	4.2
Self‐reported fever	38.6
NR	1.5
Fever measurement method (*N* = 232)	
The woman reported feeling feverish	68.5
With thermometer at the hospital (≥38°C)	19.4
With thermometer at home (≥38°C)	9.0
Other	2.2
NR	0.9
History of RTI	
No	49.6
Occasionally	31.6
Often	12.5
NR	6.3
Treatments used for current or previous RTIs	
None	5.8
Doctor‐prescribed medicine	35.8
Self‐limited (no treatment)	19.5
Herbal medicine	11.3
Over‐the‐counter drugs	4.3
NR	23.3
History of antibiotic use due to all causes	31.1
NR	8.3
Hygiene after toilet use	
Water	82.0
Toilet paper	15.1
Cloth	1.2
NR	1.7
Hand washing with water and soap after toilet use	
No	6.8
Always	49.4
Always, but soap is sometimes not available	7.8
Sometimes forget	28.5
Rarely	6.5
NR	1.0
Knowledge about RTIs	23.0
NR	2.3
Husband's history of RTI	
No	76.2
Yes	8.9
NR	14.9
Time between signs and symptoms onset and care‐seeking	
No delay (within 5 days from symptoms onset)	45.8
Up to 2 weeks	11.5
Over 2 weeks and up to 1 month	9.6
Over 1 month	29.9
NR	3.2
Reason for delay (*N* = 307)	
Unaware of symptoms’ implications	28.7
Misdiagnosed at a previous clinic	23.4
Could not afford healthcare	19.9
Could not access healthcare	11.7
Fearful or ashamed of a possible diagnosis	5.9
Felt disrespected at a previous clinic	2.6
Other	2.0
NR	16.9

*Note:* Abnormal vaginal discharge = deviating from the woman's average discharge quantity, color, consistency, or smell, for at least 2 days during the last month. Knowledge of RTIs = the women already knew about the infectious nature of RTIs and their modes of transmission.

Abbreviation: NR, not reported.

^a^ The five signs and symptoms considered were the following: abnormal vaginal discharge, vaginal itching, smell from genitals, lower abdominal pain, and fever.

When assessing the time from symptoms onset and seeking healthcare, it was found that 45.8% of participants did not experience any delay (Table 2). Conversely, delays of up to 2 weeks, up to 1 month, and over 1 month were reported by, respectively, 11.5%, 9.6%, and 29.9%. Women cited being unaware of the implications of the signs and symptoms as the most frequent reason for the delay (28.7%); however, interestingly, 23.4% of the respondents reported that they had previously gone to another clinic, where they received no further investigations or care, or they felt disrespected (another 2.6%). Issues with affording or accessing care were mentioned by 31.6%, whereas fear or shame related to the possible diagnosis were only declared by 5.9%.

### 3.6 Predictors of Delay in Care‐Seeking

Women who delayed their visit to a clinic for 1 month or longer after the appearance of signs or symptoms were more likely to also report signs and symptoms: OR 4.12 (95% CI 2.01–8.45) for abnormal vaginal discharge, 1.93 (1.25–2.98) for fever, and 2.62 (1.44–4.77) for lower abdominal pain (Table 3). Instead, delays were negatively associated with being sedentary (0.38; 0.22–0.64), with hand washing after toilet use (0.61; 0.40–0.95), and with having knowledge about RTIs (0.60; 0.31–1.00), although borderline significant.

**TABLE 3:** Odds ratios (OR) and 95% confidence intervals (CI) of delay in care‐seeking and history of reproductive tract infection (RTI), according to the selected covariates.

Covariates	Delayed care‐seeking, OR (95% CI) (*n* = 555)	History of RTI, OR (95% CI) (*n* = 568)
Abnormal vaginal discharge	4.12 (2.01–8.45)	2.29 (1.38–3.79)
Fever	1.93 (1.25–2.98)	1.56 (1.04–2.36)
Lower abdominal pain	2.62 (1.44–4.77)	0.96 (0.60–1.54)
Age ≤25 years	1.02 (0.60–1.73)	0.75 (0.47–1.22)
Illiterate	0.88 (0.52–1.47)	0.87 (0.54–1.41)
Knowledge on RTIs	0.60 (0.35–1.00)	1.28 (0.81–2.01)
Income lower than average	1.03 (0.65–1.65)	1.49 (0.97–2.29)
Sedentary	0.38 (0.22–0.64)	1.11 (0.72–1.70)
Never married	1.00 (ref. cat.)	1.00 (ref. cat.)
Husband without RTI history	1.57 (0.74–3.32)	2.02 (0.93–4.38)
Husband with RTI history	1.71 (0.90–3.24)	0.80 (0.44–1.46)
Husband with unknown RTI history	1.21 (0.64–2.30)	0.42 (0.23–0.77)
Condom use	0.81 (0.51–1.30)	0.71 (0.46–1.09)
Irregular menstruations	0.76 (0.50–1.16)	1.59 (1.07–2.35)
Sanitary pad use (vs. cloth)	0.99 (0.61–1.60)	0.63 (0.41–0.97)
Hand washing after each toilet use	0.61 (0.40–0.95)	0.88 (0.59–1.31)
Previous antibiotic use	0.75 (0.48–1.18)	1.24 (0.82–1.86)

*Note:* All signs, symptoms, and other women's characteristics were self‐reported. Delayed care‐seeking = wait of at least 30 days from signs and symptoms onset to seeking care at an official healthcare provider, for reasons other than mismanagement at a previous clinic. History of RTI = at least one self‐reported diagnosis of RTI at any point in the woman's life. Abnormal vaginal discharge = deviating from the woman's average discharge quantity, color, consistency, or smell for at least 2 days during the last month. Sedentary = women who declare doing no physical activity. Irregular menstruation = menstrual cycle lasting more than 35 days in at least 4 out of 5 cycles [21]. Condom use = always used during intercourse except when seeking a pregnancy.

### 3.7 Predictors of History of RTI

Similarly to delays, a history of RTI was also associated with signs and symptoms except lower abdominal pain: OR 2.29 (95% CI 1.38–3.79) for abnormal vaginal discharge and 1.56 (1.04–2.36) for fever. Another positive predictor was having irregular menstruations (1.59; 1.07–2.35), whereas, on the contrary, sanitary pads showed a negative association (0.63; 0.41–0.97). Surprisingly, reporting a positive history of RTI for the husband was not associated with the wife's own history of RTI (0.80; 0.44–1.46).

## 4 Discussion

This cross‐sectional study interviewed women attending clinics in multiple cities in Afghanistan and found that almost four out of five women reported at least one symptom suggestive of RTI, yet less than one fourth had knowledge regarding RTI. Delayed care‐seeking was also common, although it was sometimes caused by a mismanagement occurred at the previous clinic visit.

In women, RTIs typically manifest as lower genital tract infections, with symptoms such as abnormal vaginal discharge, itching, genital pain, and fever [22]. The WHO estimated that in low‐income countries in 2016, the prevalence of RTIs ranged from 2% for gonorrhea and syphilis to 4% for chlamydia up to 10% for trichomoniasis [23]. This high prevalence is confirmed by community studies among Afghan refugees and communities of neighboring countries [10, 24, 25], and also, at least apparently, by the high proportion of symptomatic women found in the present work. Naturally, the prevalence of signs and symptoms estimated in this study cannot be generalized to the whole female population of Afghanistan, as the sample was drawn from women presenting to clinics and not from the general population. Nevertheless, given the satisfactory response rate and that all the clinics were multi‐specialist and not only for gynecology, but the provided prevalence is also useful to gauge the needs of Afghan women who seek healthcare.

More than half of the interviewed women reported a delay in seeking gynecological care, often because they experienced some kind of mismanagement at the healthcare provider they visited prior to the clinic where they were interviewed. A seemingly counterintuitive finding, namely, that symptomatic women were more likely to delay care‐seeking, may be interpreted in light of the stigma that characterizes RTIs. Indeed, there is a widespread belief among Muslim population that RTIs are transmitted through extramarital sex [26], a misconception exhibited even by some healthcare professionals [27]. Hand washing was expectedly associated with a lower risk of delay, as was being sedentary, a finding that requires further exploration. Having knowledge on RTIs was negatively associated with delay, although with only borderline significance. One possible explanation of the limited impact of knowledge is that fear of stigma may affect the majority of women regardless of the severity of symptoms, inhibiting them from seeking help [26].

Regarding the women who reported a history of RTI, the history of RTI of their husbands was unexpectedly unrelated to this outcome. Indeed, to the women's knowledge, only a small fraction of the husbands had a history of RTI. This can be explained by two reasons. First, their partners might have not disclosed their STI diagnosis. Previous studies have shown that men are often uncomfortable disclosing an STI diagnosis to their partners [28, 29]. In a country with a high gender inequality as Afghanistan [6], men may be less compelled to disclose their diagnosis to their partners. Second, some common RTIs are not sexually transmitted, such as yeast infection and bacterial vaginosis [21]. Some of the symptoms most commonly reported by our respondents, such as vaginal discharge, are related to these diseases. On the other hand, hygiene practices are generally associated with a lower risk of RTI [4], and indeed the use of sanitary pads instead of cloth resulted negatively associated with a history of RTI in our sample.

Our study found that less than a quarter of respondents had adequate knowledge about RTIs, possibly relating to the high number of illiterates among our respondents, and that RTI knowledge could presumably protect against delays in seeking care. This is consistent with a 2004 study in Kabul, Afghanistan, which found that only 24% of respondents had knowledge on RTIs and that formal schooling was one of the determinants of STIs awareness [9]. Previous studies in the United States and Brazil showed that stigma‐associated fear and anxiety led to decreased willingness of having RTI tests [16, 20]. Although studies on the extent of such stigma among Afghan women are lacking, it was explored among midwives, one quarter of which reported hesitancy to associate with RTI‐infected women [15].

Concerning possible interventions, it was shown that stigma can be reduced through awareness programs [30], which should be developed considering the current societal norms and gender roles. Furthermore, the health system should be strengthened, and public‐private partnership, already employed in the management of other diseases, should also be extended to RTIs in Afghan women in order to reduce the care‐seeking delay [31].

Health system barriers were cited as reasons for delayed care‐seeking by one third of the respondents, also an expected result. Afghanistan, as a low‐income country with an ongoing humanitarian crisis, has a limited number of healthcare facilities: there are only 0.3 physicians and 0.4 nurses and midwives per 1000 people, lower than the threshold determined by the WHO (4.45 doctors, nurses, and midwives per 1000 people) [32, 33]. Since the 2021 government takeover, many healthcare professionals have left the country, and the healthcare system is paralyzed in many areas, particularly in the villages [34], where about 20% of our respondents lived. It is also important to note that in Afghanistan, the gender roles discourage women from seeking care at medical facilities [26]. Restrictions of women's movement without their kin or husbands (mahram), as well as the norm banning women to be examined by male doctors, have all led to reduced access to healthcare [6, 34].

Our study offers valuable data exploring RTI health seeking behavior of Afghan women after Taliban takeover for stakeholders both nationally and internationally to provide life‐saving reproductive health services for Afghan women to insure timely diagnose and management of RTI. In addition, as our study highlights, to address a lack of reproductive health knowledge, health education interventions through health literacy promotion polices can have a detrimental impact on reproductive health of women.

### 4.1 Strengths and Limitations

Our study is the first to describe RTI health seeking behavior in a large sample of Afghan women post‐Taliban takeover. To maximize the insight of the data, the data collection was conducted in a culturally sensitive manner by our interviewers. We also translated the questionnaire into two native languages in order to improve comparability. However, our study is not without limitations. Its design relied on self‐reported data, hence the possibility of recall bias. This is particularly possible for husband‐related data, as we did not ask the husbands directly. To reduce the possibility of bias, particularly in terms of symptoms reporting, we limited the timeframe for the reported signs and symptoms to 1 month prior to the interview. One further limitation of this study is that it was conducted in clinics, where the majority of patients are assumed to have good access to healthcare and the knowledge to seek care from specific providers. Therefore, the findings may not fully represent women from very low‐resource villages or those who typically rely on natural remedies such as herbalism or traditional medicine for healthcare needs.

## 5 Conclusion

The present study interviewed for the first time a large sample of women from multiple cities in Afghanistan. The majority of women who seek care at multi‐specialist clinics in Afghanistan present with signs and symptoms likely related to RTIs and with over 1 month of delay since the symptoms onset. Furthermore, knowledge about RTI transmission is severely lacking, suggesting that healthcare providers should inform the population about the standard care pathway for such cases, adopt a multi‐dimensional approach which takes into account the cultural background of the women and, in general, promote higher awareness about RTIs among the general population, aiming to reduce the stigma associated with them.

## Author Contributions

**Cecilia Acuti Martellucci**: formal analysis, conceptualization, methodology, writing – review and editing, validation, data curation, software. **Nooria Mohammady**: writing – review and editing, investigation, methodology, project administration, data curation, writing – original draft. **Fawzia Negin**: investigation, writing – review and editing, conceptualization. **Sayed Hamid Mosavi**: conceptualization, writing – review and editing, supervision. **Adriana Viola Miranda**: formal analysis, software, methodology. **Husna Safa**: writing – review and editing, investigation, data curation. **Bibi Qudsia Qasimi**: writing – review and editing, investigation, data curation. **Khaterah Mosavi**: writing – review and editing, investigation. **Basira Bek**: writing – review and editing, investigation, methodology. **Alisina Azimi**: data curation, writing – review and editing, investigation. **Zahra Annabi**: investigation, writing – review and editing, conceptualization. **Saddiqa Noorzai**: investigation, writing – review and editing. **Aabidullah Rahimee**: writing – review and editing, investigation. **Taha Balaghat**: investigation, writing – review and editing. **Fatema Rezaie**: investigation, writing ‐ review and editing. **Fardına Temory**: investigation, writing – review and editing. **Mirwais Ramozi**: investigation, writing – review and editing, data curation. **Mosè Martellucci**: methodology, formal analysis, writing – review and editing. **Rahila Bek**: investigation, writing – review and editing. **Shabana Noroozi**: investigation, writing – review and editing. **Madina Niro**: writing – review and editing, investigation. **Husna Sultani**: investigation, writing – review and editing. **Palwasha Farooqi**: investigation, writing – review and editing. **Ehsan Shayan**: investigation, writing – review and editing. **Mohammadgul Zhwand**: writing – review and editing, conceptualization. **Qasem Rezaee**: conceptualization, writing – review and editing. **Farzana Torgani**: investigation, writing – review and editing. **Bibi Shakiba Hussaini**: investigation, writing – review and editing. **Shaqaiq Akhtiyari**: conceptualization, writing ‐ review and editing. **Farah Qaderi**: visualization, resources, methodology, writing – review and editing. **Shohra Qaderi**: supervision, project administration, validation, conceptualization, methodology, writing – review and editing, writing – original draft, data curation.

## Ethics Statement

Ethical approval for the study was obtained from the Afghanistan National Charity Organization for Special Diseases (Approval code: AF, ANCOSD, HERC, 09, September 16th 2022), which also approved the verbal nature of consent. All study procedures were conducted in accordance with the Declaration of Helsinki on medical research.

## Conflicts of Interest

The authors declare no conflicts of interest.

## Supporting information




**Table S1**: Characteristics of women's husbands (*N* = 423).
**Table S2**: Gynecological characteristics of the women (*N* = 601).
**Table S3**: Reproductive history of the women (*N* = 440).
**Table S4**: Characteristics of women with suspected infertility (*N* = 109).

## Data Availability

The dataset and the questionnaire used during the current study are available from the corresponding author on reasonable request.
