# Supplementary material for: Understanding Care‐Seeking Behavior for Reproductive Tract Infections Among Afghan Women: A Cross‐Sectional Study
Source: Public Health Chall. 2025 Jun 18;4(2):e70072. doi: 10.1002/puh2.70072 (PMC12174964; doi:10.1002/puh2.70072)
Supplement: Supplementary file 1 — Table S1: Characteristics of women's husbands (N = 423). Table S2: Gynecological characteristics of the women (N = 601). Table S3: Reproductive history of the women (N = 440). Table S4: Characteristics of women with suspected infertility (N = 109). [file PUH2-4-e70072-s001.docx]

**Supplementary Tables**

**Table S1.** Characteristics of women’s husbands (N=423).

| Husbands’ characteristics | Proportions  (%)* |
| --- | --- |
|  |  |
| Age in years, mean (SD) | 40.5 (12.3) |
| Age |  |
| 20-25 | 6.2 |
| 26-35 | 34.3 |
| 36-45 | 31.4 |
| >45 | 28.1 |
|  |  |
| Unemployed (N=448) | 29.2 |
|  |  |
| History of dysuria | 12.3 |
| NR | 6.2 |
|  |  |
| History of RTI |  |
| No | 76.2 |
| Yes | 8.9 |
| Unknown | 14.9 |
|  |  |

* Percentages unless otherwise specified.

NR = not reported. RTI = reproductive tract infection.

**Table S2.** Gynecological characteristics of the women (N=601).

| Characteristics | Proportions  (%)* |
| --- | --- |
|  |  |
| Age at menarche in years, mean (SD) | 13.1 (1.9) |
| Age at menarche |  |
| <12 | 13.4 |
| 12-13 | 40.1 |
| 14-15 | 38.8 |
| >15 | 6.0 |
| NR | 2.0 |
|  |  |
| Age at menopause in years, mean (SD) | 46.0 (5.4) |
| Age at menopause |  |
| <48 | 6.2 |
| ≥48 | 4.5 |
| No menopause | 89.3 |
|  |  |
| Average bleeding duration in days, mean (SD) | 6.0 (1.7) |
| Average bleeding duration |  |
| <5 | 15.0 |
| ≥5 | 81.2 |
| NR | 3.8 |
|  |  |
| Menstruation care |  |
| Sanitary pads | 65.0 |
| Cloths or other care | 32.3 |
| NR | 2.7 |
|  |  |
| Irregular menstruations | 32.0 |
| NR | 2.3 |
|  |  |
| Painful menstruation | 77.0 |
| NR | 3.3 |
|  |  |

* Percentages unless otherwise specified.

NR = not reported (equals 0.0% when absent).

**Table S3.** Reproductive history of the women (N=440).

| Characteristics | Proportions  (%)* |
| --- | --- |
|  |  |
| Age at marriage in years, mean (SD) | 19.5 (4.2) |
| Age at marriage |  |
| <17 | 21.4 |
| 17-20 | 45.7 |
| 21-24 | 21.1 |
| >24 | 10.0 |
| NR | 1.8 |
|  |  |
| Age at first delivery in years, mean (SD) | 21.1 (3.7) |
| Age at first delivery |  |
| <17 | 6.8 |
| 17-20 | 32.0 |
| 21-24 | 31.4 |
| >24 | 13.9 |
| NR | 15.9 |
|  |  |
| Age at last delivery in years, mean (SD) | 30.9 (6.8) |
| Age at last delivery, % |  |
| <17 | 0.0 |
| 17-20 | 3.6 |
| 21-24 | 11.4 |
| >24 | 63.4 |
| NR | 21.6 |
|  |  |
| Number of children alive, mean (SD) | 3.8 (2.5) |
| Number of children alive |  |
| 0 | 3.0 |
| 1-2 | 21.0 |
| 3-4 | 19.0 |
| >4 | 22.0 |
| NR | 34.9 |
|  |  |
| Number of vaginal deliveries, mean (SD) | 3.9 (2.5) |
| Number of vaginal deliveries |  |
| 0 | 1.6 |
| 1-2 | 23.8 |
| 3-4 | 22.3 |
| >4 | 25.0 |
| NR | 27.3 |
|  |  |
| Number of C-sections, mean (SD) | 0.4 (0.8) |
| Number of C-sections |  |
| 0 | 64.3 |
| 1-2 | 17.5 |
| 3-4 | 2.5 |
| NR | 15.7 |
|  |  |
| Number of spontaneous abortions, mean (SD) | 1.2 (1.6) |
| Number of spontaneous abortions |  |
| 0 | 23.9 |
| 1-2 | 25.9 |
| 3-4 | 6.1 |
| >4 | 2.0 |
| NR | 42.1 |
|  |  |
| At least one stillbirth | 10.2 |
| NR | 2.3 |
|  |  |
| At least one ectopic pregnancy | 3.0 |
| NR | 3.6 |
|  |  |
| At least one twin delivery | 4.3 |
|  |  |
| Months between last two childbirths, mean (SD) | 34.1 (19.7) |
| Months between last two childbirths |  |
| <13 | 8.9 |
| 13-24 | 27.0 |
| 25-36 | 16.8 |
| >36 | 20.0 |
| NR | 27.3 |
|  |  |
| Months breastfeeding last childbirth, mean (SD) | 17.5 (8.9) |
| Months breastfeeding last childbirth |  |
| 0 | 4.6 |
| 2-12 | 23.9 |
| 13-24 | 50.4 |
| >24 | 7.0 |
| NR | 14.1 |
|  |  |
| Hypertension or pre-eclampsia at last pregnancy | 21.8 |
| No | 65.9 |
| Did not check | 7.3 |
| Had no screening available | 3.0 |
| NR | 2.0 |
|  |  |
| Currently pregnant | 14.8 |
|  |  |
| Place of delivery |  |
| Always at the hospital | 48.9 |
| Sometimes at the hospital and sometimes at home | 20.0 |
| Always at home | 15.2 |
| Other | 2.0 |
| NR | 13.9 |
|  |  |

* Percentages unless otherwise specified.

NR = not reported (equals 0.0% when absent).

**Table S4.** Characteristics of women with suspected infertility (N=109).

| Characteristics | Proportions  (%)* |
| --- | --- |
|  |  |
| Overall failure to get pregnant, (N=601) | 18.1 |
|  |  |
| Mean age in years, mean (SD) | 34.4 (10.1) |
|  |  |
| Sought physician’s help, % |  |
| Yes, more than one physician | 54.1 |
| Yes, one physician | 31.2 |
| Not yet | 11.9 |
| NR | 2.8 |
|  |  |
| Infertility diagnosis, % | 31.2 |
| NR | 2.8 |
|  |  |
| In vitro fertilization, % | 3.7 |
| NR | 15.5 |
|  |  |

* Percentages unless otherwise specified.

NR = not reported (equals 0.0% when absent).
